# Multiplex profiling identifies clinically relevant signalling proteins in an isogenic prostate cancer model of radioresistance

**DOI:** 10.1038/s41598-019-53799-7

**Published:** 2019-11-22

**Authors:** S. Inder, M. Bates, N. Ni Labhrai, N. McDermott, J. Schneider, G. Erdmann, T. Jamerson, V. A. Belle, A. Prina-Mello, P. Thirion, P. R. Manecksha, D. Cormican, S. Finn, T. Lynch, L. Marignol

**Affiliations:** 1 Translational Radiobiology and Molecular oncology, Applied Radiation Therapy Trinity, Trinity Translational Medicine Institute (TTMI), Trinity College Dublin, Dublin, Ireland; 2 Department of Urology, St James’s Hospital, Dublin, Ireland; 3 NMI TT Pharmaservices, Berlin, Germany; 4 Department of International Health, Mount Sinai School of Medicine, New York, USA; 5 Laboratory for Biological Characterization of Advanced Materials (LBCAM), Trinity Translational Medicine Institute (TTMI), AMBER centre at CRANN Institute, Trinity College Dublin, Dublin, Ireland; 6 Department of Clinical Medicine, School of Medicine, Trinity College Dublin, Dublin, Ireland; 7 St Luke’s Radiation Oncology Network, St James’s Hospital, Dublin, Ireland; 8 Department of Surgery, Trinity College Dublin, Dublin, Ireland; 9 Department of Histopathology, St James’s Hospital, Dublin, Ireland

**Keywords:** Cancer, Radiotherapy

## Abstract

The exact biological mechanism governing the radioresistant phenotype of prostate tumours at a high risk of recurrence despite the delivery of advanced radiotherapy protocols remains unclear. This study analysed the protein expression profiles of a previously generated isogenic 22Rv1 prostate cancer model of radioresistance using DigiWest multiplex protein profiling for a selection of 90 signalling proteins. Comparative analysis of the profiles identified a substantial change in the expression of 43 proteins. Differential PARP-1, AR, p53, Notch-3 and YB-1 protein levels were independently validated using Western Blotting. Pharmacological targeting of these proteins was associated with a mild but significant radiosensitisation effect at 4Gy. This study supports the clinical relevance of isogenic *in vitro* models of radioresistance and clarifies the molecular radiation response of prostate cancer cells.

## Introduction

Prostate tumours not controlled by radiation therapy^1^ may present with radiation protective biological characteristics^2^ whose pre-treatment identification has the potential to predict treatment outcomes and initiate the development of novel, more aggressive, treatment options. Isogenic models of radioresistance are emerging as clinically-relevant models for the study of these tumours characteristics^3^. This approach has been particularly useful in the characterisation of the radiation-induced DNA damage response^4,5^. But all identified capabilities or hallmarks of cancer cells can help explain the radiobiological response of tumours^3^. As a result, the signalling pathways known to regulate several cancer hallmarks, such as p53 and Notch^6^, may be key to the regulation of radioresistant cancer cells fate.

While the role of p53 in the increased survival of prostate cancer cells to fractionated radiation^7^, increased cell proliferation^8^ and treatment outcomes^9^ in patients with locally recurrent prostate carcinoma after radiation therapy has been documented, implications of the Notch pathway in the radiation response^10^ is not reported in prostate tumours. The Notch pathway is implicated in angiogenesis^11,12^ and has been proposed to facilitate prostatic tumourigenesis^13^, influence the outcome of anti-cancer hormonal^14,15^ and docetaxel treatments^16^ and may be particularly involved in the development of prostate cancer in men with high body mass index^17,18^. Investigation into the regulation of this pathway indicates a possible cross talk with the YB-1 pathway^19,20^. YB-1 is a multifunctional protein whose expression increases with prostate cancer progression and is predictive of recurrence following surgery^21^. It is involved in both the transcriptional and translational regulation of gene expression, and controls almost all DNA and mRNA dependent processes in the cell such as cellular differentiation, proliferation and stress response^22^.

In prostate cancer, exposure to fractionated radiation progressively selected for a 22Rv1 prostate carcinoma cell population enriched in S-phase cells, less susceptible to DNA damage, radiation-induced apoptosis and acquired enhanced migration potential, when compared to wild type and aged-matched control 22Rv1 cells^23^. These enhanced radioprotective oncogenic properties, also observed in isogenic models of other disease sites^3^, were associated with an altered miRNA profile common to that of 22Rv1 cells exposed to hypoxia, a known factor associated with radioresistance^24,25^. This study aimed to further establish the clinical relevance of the model and identify candidate markers of radioresistance for this disease. Ninety proteins associated with the cancer hallmarks, the Notch and the YB-pathways were selected to generate a custom multiplex protein expression profile of radioresistant (RR-22Rv1) and radiosensitive (WT-22Rv1) isogenic prostate cancer cells. Independent validation of differentially expressed PARP-1, p53 and the androgen receptor strengthens the clinical relevance of the model and suggests a role for the Notch-3 intracellular domain (N3ICD) in the radioresponse of these cells. Pilot analysis in pre-treatment biopsies of prostate cancer patients treated with radiation therapy for the first time implicates the YB-1 protein in treatment failure.

## Results

### Radiation response of 22Rv1 isogenic cells

**Figure 1:**
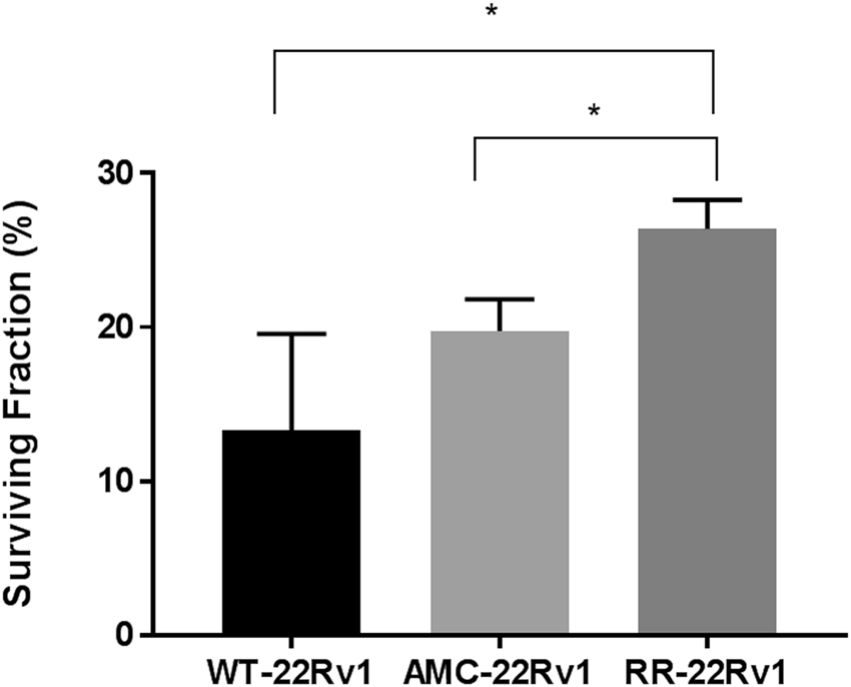
Radiation response of isogenic 22Rv1 cells. The clonogenic survival of wild type (WT), age-matched controls (AMC) and radioresistant (RR) 22Rv1 prostate cancer cells following a single dose of 4 Gy radiation is presented. N = 4; p < 0.05.

The change in the radiation response of 22Rv1 cells exposed to 30 × 2Gy- dose fractions (RR-22Rv1), compared to age-matched (AMC-22Rv1) and wild type (WT-22Rv1) cells was confirmed using clonogenic assays. The clonogenic survival of each cell line treated with a 4 Gy single dose and their corresponding unirradiated controls is presented in Fig. 1. With a mean survival of 26.4% ± 0.01, RR-22Rv1 cells were significantly more radioresistant than both AMC-22Rv1 (18.4% ± 0.01) and WT-22Rv1 (10.31% ± 0.01) cells. AMC-22Rv1 showed a non-significant trend towards increased radioresistance, when compared to WT-22Rv1 cells.

### Protein profile of the 22Rv1 cells panel

**Figure 2:**
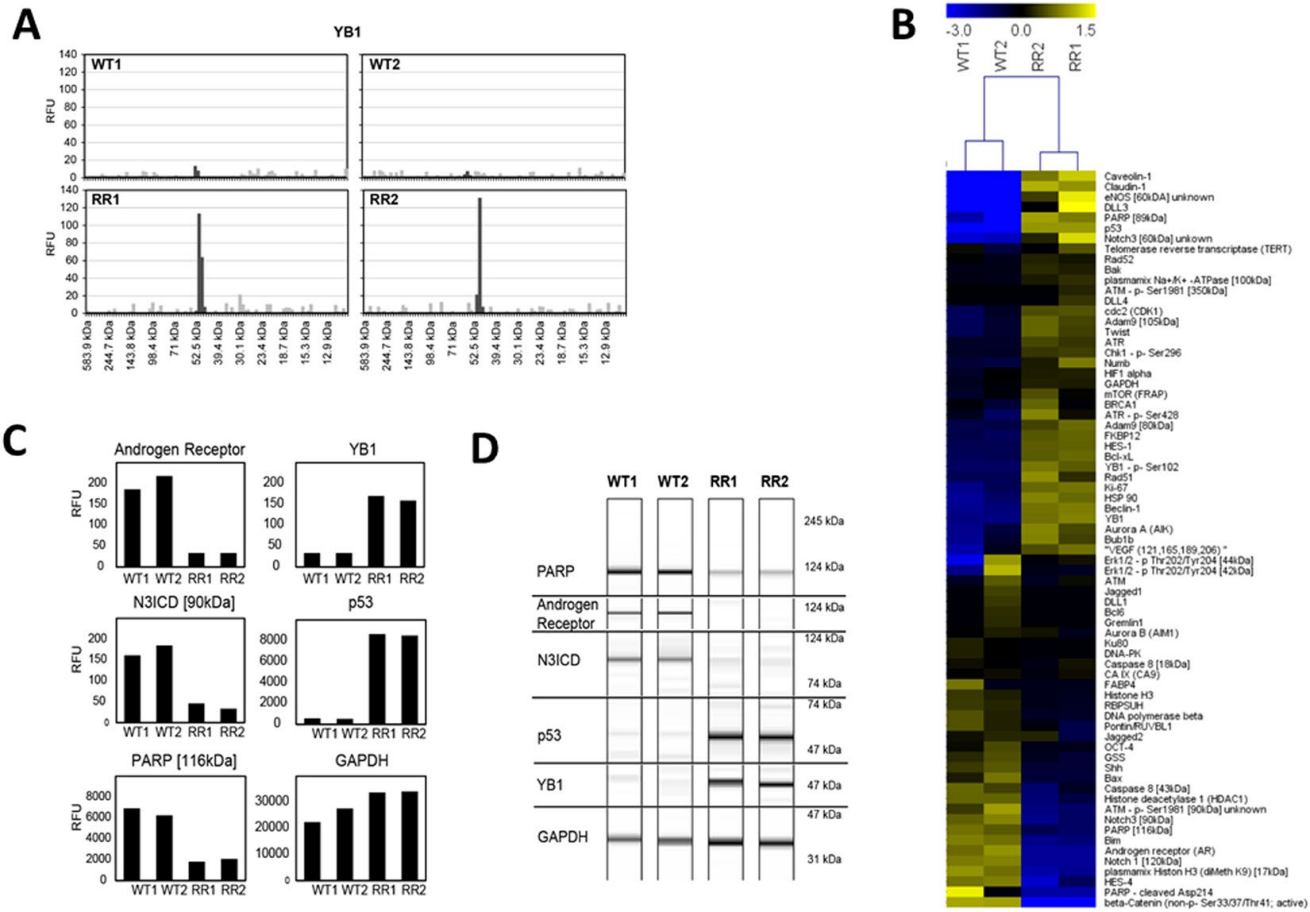
DigiWest protein profiling of wildtype (WT) and radioresistant (RR) 22Rv1 cell lines. (**A**) Typical representation of DigiWest raw data showing signals for each molecular weight group. Peak signals at the correct molecular weight are marked and quantified in Relative Fluorescence Units (RFU). (**B**) Heat map of Log2 transformed DigiWest data in WT and RR cell lines. Hierarchical Clustering was performed using complete linkage and Eucledian distance, applying the Multi Experiment Viewer (MeV) software. (**C**) Bar graphs for DigiWest data from selected proteins in WT vs RR cells. (**D**) Representation of DigiWest data as Western Blot mimics.

The protein profiles of the isogenic radioresistant cell line (RR-22Rv1) and its parent WT-22Rv1 cell lines were generated for a custom 90 proteins (Fig. 2). Comparison of these profiles identified significant modifications in expression levels for 23 proteins (14 up, 9 down), non-significant trends for 41 (20 up, 21 down) (Table 1), and failed protein detection for 23 (Table 2). The upregulated proteins were associated with the promotion of cell survival, proliferation and invasion (p53, ATR, FKBP12, Bak, Bcl-xL, Beclin-1, Calveolin-1, plasmamix Na+/K+ -ATPase, Claudin-1, Chk1-p-Ser296), the activation of Notch-3 (Notch3 intracellular domain (N3ICD), HES-1) and YB-1 (YB-1, YB-1-p-Ser1-2) signalling. The down regulated proteins were associated with genome instability (plasmamix Histon H3 (diMeth K9), Histone H3), the regulation of tumour growth (Androgen receptor, beta-catenin, Bim, Shh), DNA repair (PARP-1) and Notch signaling (Notch 1 intracellular domain (N1ICD), RBPSUH).

**Table 1:** Differential protein expression patterns in RR-22Rv1 compared to WT-22Rv1 cell samples.

	Analyte	Pathway/Function	Mean WT	Mean RR	Relationship between RR and WT	T.Test (Log 2 Values)
**Proteins with a significant increase in expression in RR-22Rv1 compared to WT-22Rv1**						
1	plasmamix Na+/K+−A TPase [100 kDa]	Intracellular pumps	178.5	258.0	+	0.0305
2	Notch3 intracellular domain [(N3ICD) 90 kDa]	Notch Signalling/Development	173.0	1642.5	+	0.0386
3	ATR	Sense DNA damage/Cell cycle arrest	33.3	63.5	+	0.0389
4	Bak	Apoptosis	33.3	48.0	+	0.0364
5	Bcl-xL	Apoptosis	1176.5	3718.0	+	0.0011
6	Beclin-1	Autophagy	111.0	560.0	+	0.0193
7	Caveolin-1	Vesicular trafficking/Cell adhesion/Apoptosis	33.3	3459.0	+	0.0217
8	Chk1 - p- Ser296	DNA Damage	33.3	61.0	+	0.0173
9	Claudin-1	Cytoskeletal regulation/Adhesion	73.5	4148.5	+	0.0031
10	FKBP12	TGF-beta/Smad signaling	6081.0	18052.0	+	0.0279
11	HES-1	Transcription Regulation (Supression)	33.3	102.0	+	0.0056
12	p53	Cell cycle control/Tumor suppressor	550.5	8581.5	+	0.0052
13	YB1	PI3K/Akt Signaling	33.3	164.5	+	0.0133
14	YB1 - p- Ser102	Transcription factor, RNA metabolism, Protein Synthesis	33.3	122.0	+	0.0282
	**Analyte**	**Pathway/Function**	**Mean WT**	**Mean RR**	**Relationship between RR and WT**	**T**.**Test (Log 2 Values)**
**Proteins with a significant decrease in expression in RR-22Rv1 compared to WT-22Rv1**						
1	plasmamix Histon H3 (diMeth K9) [17 kDa]	Chromatin regulation/Epigenetics	40142.0	6217.0	−	0.0059
2	Notch 1 intracellular domain [120 kDa]	Notch Signalling	201.5	33.3	−	0.0044
3	PARP-1 [116 kDa]	Apoptosis, DNA damage	6580.5	1944.5	−	0.0078
4	Androgen receptor (AR)	Nuclear Receptor	202.0	33.3	−	0.0281
5	beta-Catenin (non-p- Ser33/37/Thr41; active)	WNT Signalling	3070.0	33.3	−	0.0021
6	Bim	Apoptosis	722.5	148.0	−	0.005
7	RBPSUH	Development	2207.5	1355.0	−	0.0136
8	Shh	Development	73.5	33.3	−	0.0494
9	Histone H3	Chromatin regulation/Epigenetics	1133292.5	721462.5	−	0.0452
	**Analyte**	**Pathway/Function**	**Mean WT**	**Mean RR**	**Relationship between WT and RR**	**T**.**Test (Log 2 Values)**
**Proteins with a non- significant increase in expression in RR-22Rv1 compared to WT-22Rv1**						
1	ATM - p- Ser1981 [350 kDa]	Cell cycle arrest/DNA Repair/Apoptosis	33.3	37.7	+	0.5
2	ATR - p- Ser428	Sense DNA damage/Cell cycle arrest	69.0	164.0	+	0.138
3	Aurora A (AIK)	Cell Cycle Checkpoint	47.2	166.5	+	0.1106
4	BRCA1	DNA Damage	40.7	65.5	+	0.2946
5	Bub1b	Cell cycle/Checkpoint	209.5	679.5	+	0.1633
6	cdc2 (CDK1)	Cell cycle/Checkpoint	961.0	2321.0	+	0.1355
7	DLL3	Development	33.3	663.0	+	0.1192
8	DLL4	Development	33.3	39.7	+	0.5
9	GAPDH	Glucose metabolism	25021.5	33990.5	+	0.2037
10	HIF1 alpha	Protein translation	41.2	56.5	+	0.3317
11	HSP 90	Protein folding/Chaperones	312.0	1470.0	+	0.0636
12	Ki-67	Proliferation/Tumor marker	681.0	2752.0	+	0.0943
13	mTOR (FRAP)	mTOR Signalling	565.0	884.5	+	0.171
14	Numb	Development	168.5	397.5	+	0.1339
15	Rad51	DNA Damage	33.3	106.5	+	0.1076
16	Rad52	DNA Damage	300.5	398.5	+	0.1806
17	Telomerase reverse transcriptase (TERT)	Replication	47.2	68.0	+	0.4206
18	Twist	Transcription	124.0	316.0	+	0.0603
19	VEGF (121,165,189,206)	Growth Factors/Cytokines	69.2	210.0	+	0.267
20	Adam9 [105 kDa]	Cytoskeletal Signaling	101.0	263.0	+	0.0762
	**Analyte**	**Pathway/Function**	**Mean WT**	**Mean RR**	**Relationship between WT and RR**	**T**.**Test (Log 2 Values)**
**Proteins with a non-significant decrease in expression in RR-22Rv1 compared to WT-22Rv1**						
1	Erk1/2 - p Thr202/Tyr204 [42 kDa]	MAPK Signalling	817.0	512.0	−	0.716
2	Caspase 8 [43 kDa]	Apoptosis	123.0	47.2	−	0.1704
3	OCT 4	Stem Cell Marker	52.0	33.3	−	0.1919
4	ATM	Cell cycle arrest/DNA Repair/Apoptosis	50.7	41.2	−	0.7351
5	Aurora B (AIM1)	Cell Cycle Checkpoint	382.0	334.5	−	0.5505
6	Bax	Apoptosis	77.0	33.3	−	0.1403
7	Bcl6	Lymphocytes Signalling	38.7	33.3	−	0.5
8	CA IX (CA9)	Hypoxia Biomarker	80.0	73.5	−	0.5744
9	DLL1	Development	40.2	33.3	−	0.5
10	DNA polymerase beta	DNA Damage	1857.5	1008.0	−	0.1013
11	DNA-PK	DNA Damage	98.0	83.0	−	0.2625
12	FABP4	Metabolism	51.7	33.3	−	0.5
13	Gremlin1	Oxidative Stress/Antioxidant	38.2	33.3	−	0.5
14	GSS	Oxidative Stress	255.0	134.0	−	0.0511
15	HES-4	Transcription Regulation	295.5	56.7	−	0.156
16	Histone deacetylase 1 (HDAC1)	Chromatin regulation/Epigenetics	140.0	42.2	−	0.1096
17	Jagged1	Development	41.7	33.3	−	0.5
18	Jagged2	Development	69.5	52.7	−	0.5304
19	Ku80	DNA Damage	7350.5	6191.0	−	0.2563
20	PARP - cleaved Asp214	Apoptosis	231.0	33.3	−	0.1993
21	Pontin/RUVBL1	Chromatin remodeling, regulating gene transcription	216.5	118.5	−	0.1912

The relationship to radioresistance is marked with a (+) if expression was increased in RR− compared to WT− 22Rv1 cells. A decrease in expression is marked with a (−).

**Table 2:** List of analysed Proteins that were below detection levels.

	Protein	Pathway/Function
1	eNOS [140 kDa]	PI3K/Akt Signaling
2	Notch 1 [300 kDa]	Notch Signalling
3	Bad	Apoptosis
4	Bcl2	Apoptosis
5	CD133	MAPK signaling/Akt signaling
6	CD44	Stem Cell Marker
7	Chk2	Cell Cycle/Checkpoint
8	Cox2	NF-kB signaling
9	Dicer1	Protein Translation
10	EGFR (ErB-1, HER1)	Tyrosine kinase/Adaptor
11	Eotaxin	Inflammatory response
12	FoxO3a - p- Ser413	PI3K/Akt Signaling
13	Notch 1 - cleaved Val1744	Notch Signalling
14	Notch 2	Notch Signalling
15	P-Glycoprotein (MDR1, ABCB1)	Metabolism
16	PTEN - p- Ser380	Metabolism/Cell Cycle
17	Puma	Modulator of Apoptosis
18	Rad50	DNA Damage
19	SFRP2	Wnt signaling
20	Sox2	Stem Cell Marker
21	Stathmin 1	Cytoskeletal signaling
22	Survivin - p- Thr34	Apoptosis
23	VE-Cadherin	Adhesion
24	VEGF-A	Angiogenesis

**Figure 3:**
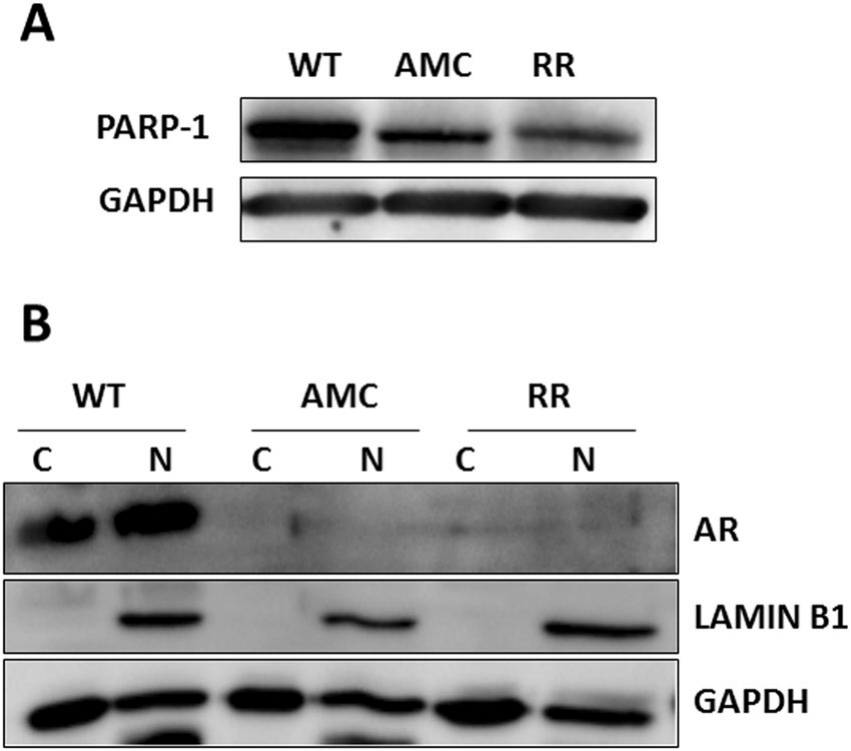
Representative cropped immunoblots of wild type (WT), age-matched controls (AMC) and radioresistant (RR) 22Rv1 prostate cancer cells. The detection levels of (**A**) PARP-1 in whole cell lysates, and (**B**) the androgen receptor (AR) in the nuclear (N) and cytoplasmic (C) protein fractions are presented. GAPDH or Lamin B1 were used as cytoplasmic and nuclear loading controls respectively. The full length blots are presented in Supplementary Figs. 1A,B and 2A,B.

Determination of the expression levels of PARP-1 and the androgen receptor in independent sets of WT-22Rv1, AMC-22Rv1 and RR-22Rv1 validated the decreased expression of these two proteins in cells with a radioresistant phenotype (Fig. 3).

### Validation of the p53 expression profile

**Figure 4:**
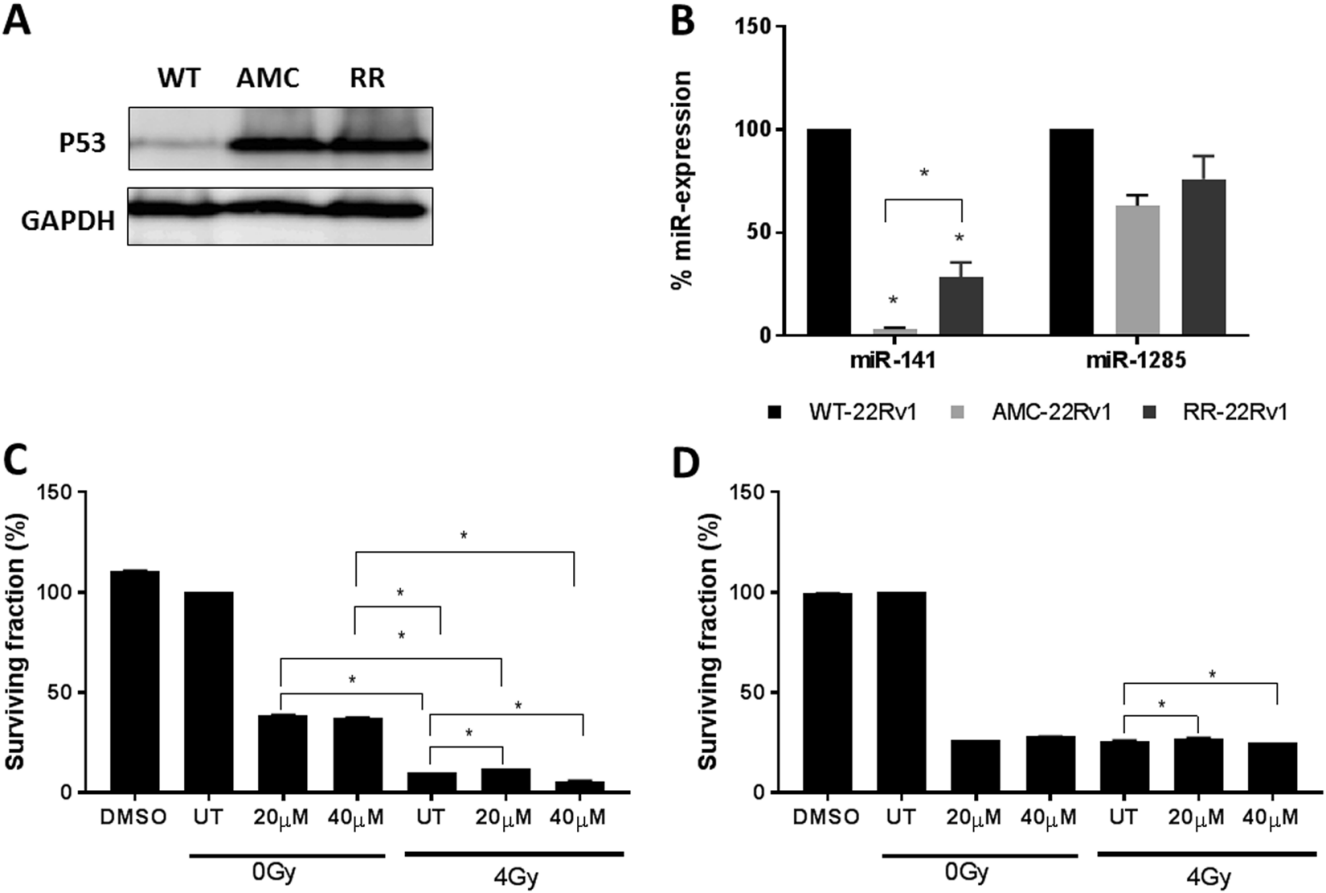
Evaluation of p53 as a marker of radioresistance. (**A**) Representative cropped immunoblots for the detection of p53 in wild type (WT), age-matched controls (AMC) and radioresistant (RR) 22Rv1 prostate cancer cells. GAPDH was used as the loading control. Full length blots are presented in Supplementary Fig. 3A,B. (**B**) miR-141 and miR-1285 expression profiles in WT-, AMC- and RR-22Rv1 prostate cancer cell lines was determined by qPCR. MiR-141 and miR-1285 expression levels for each cell line were normalised to the endogenous control miR-16 and calibrated to that of WT-22Rv1 cells to establish the relative change in microRNA expression (% miR-expression). Results are expressed as mean +/− SD, N = 3. Clonogenic survival of (**C**) WT-22Rv1 and (D) RR-22Rv1 treated with Pifithrin-α (20 µM, 40 µM, 2hrs) alone or in combination with a single dose of 4 Gy radiation. The clonogenic survival of untreated cells was used as a control. UT: untreated controls, *p < 0.05.

The expression levels of the p53 protein were confirmed to increase in radioresistant compared to wild type cells in independent cell lysates (Fig. 4A). The expression levels of the p53 pathway regulatory miRNAs, miR-141 and miR-1285 were measured by qPCR in the cell lines. While expression levels of miR-141 were significantly downregulated in RR-22Rv1 and AMC-22Rv1 compared to WT-22Rv1 cells, no significant change in miR-1285 was observed (Fig. 4). The commercially available p53 inhibitor Pifithrin-α (PFT-α) was next evaluated for its ability to affect the clonogenic survival of the cell lines. WT and RR-22Rv1 cells were treated with two separate concentrations of PFT-α (20 µM, 40 µM, 2 h) alone or in combination with radiation (4 Gy single dose) (Fig. 4). The surviving fractions of WT-22Rv1 cells treated with either 20 µM (38.7 ± 0.04) or 40 µM (37.5% ± 0.03) PFT-α alone were significantly higher than those of cells treated with radiation alone (10.31% ± 0.01) (P < 0.05). The combination of PFT-α and 4 Gy resulted in a significant reduction in surviving fraction when compared to PFT-α alone at both 20 µM (12.2% ± 0.02) and 40 µM (5.87% ± 0.01) (p < 0.05). The surviving fraction of cells exposed to the combined treatment was significantly reduced at 40 µM (p < 0.05), indicating a very small radiosensitising effect of PFT-α at 40 µM. RR-22Rv1 cells were significantly more sensitive to PFT-α than WT-22Rv1 at both concentrations alone or in combination with radiation (p < 0.05). The surviving fraction of RR-22Rv1 cells (22% ± 0.1) treated with either PFT-α concentration or 4 Gy alone were not significantly different. In response to the combination of PFT-α and 4 Gy, a very small but significant radiosensitising effect was observed as a reduction in clonogenic survival at both concentrations (p < 0.05).

### Validation of the Notch-3 expression profile

**Figure 5:**
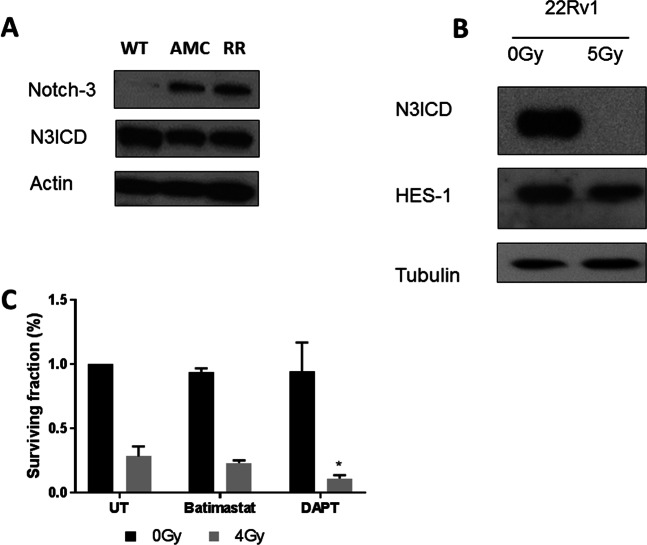
Evaluation of Notch-3 as a marker of radioresistance. (**A**) Representative cropped immunoblots of Notch-3 and its intracellular domain in wild type (WT), age-matched controls (AMC) and radioresistant (RR) 22Rv1 prostate cancer cells. The full length blots are presented in Supplementary Fig. 4. (**B**) The detection levels of the Notch-3 intracellular domain (N3ICD) and HES-1 in the protein fractions of untreated and 5Gy-irradiated WT-22Rv1 cells are presented. Actin or Tubulin were used as loading controls. The full blots are presented in Supplementary Fig. 5A–C. (**C**) Clonogenic survival of RR-22Rv1-cells treated with the Notch-inhibitors Batimastat or DAPT (60 µM, 24hrs), alone or in a combination with a 4 Gy single radiation dose. *p < 0.05.

The independent validation of the expression of the Notch-3 receptor indicated strong expression of the cleaved intracellular domain of the Notch-3 receptor (N3ICD) in WT-22Rv1, AMC-22Rv1 and RR-22Rv1 cells. The expression of the full-length receptor appeared to increase in AMC-22Rv1 and RR-22Rv1 cells (Fig. 5A). Evaluation of the cellular localization of N3ICD following radiation exposure (5 Gy) identified a loss of expression in irradiated WT-22Rv1 prostate cancer cells, but maintained HES-1 expression (Fig. 5B). Treatment of WT-22Rv1 and RR-22Rv1 cells with two commercially available Notch inhibitors 3,5-Difluorophenacetyl)-L-alanyl]-S-phenylglycine t-butyl ester (DAPT) and Batimastat (60 µM) failed to significantly decrease clonogenic survival when compared to untreated controls. When the inhibitors were combined with a single 4 Gy radiation dose, a mild radiosensitising effect was observed in RR-22Rv1 treated with DAPT, with a reduction in clonogenic survival from 28.3% ± 4.4 (radiation alone) to 11.1% ± 1.1 (p < 0.05) (Fig. 5C).

### Validation of the YB-1 expression profile

**Figure 6:**
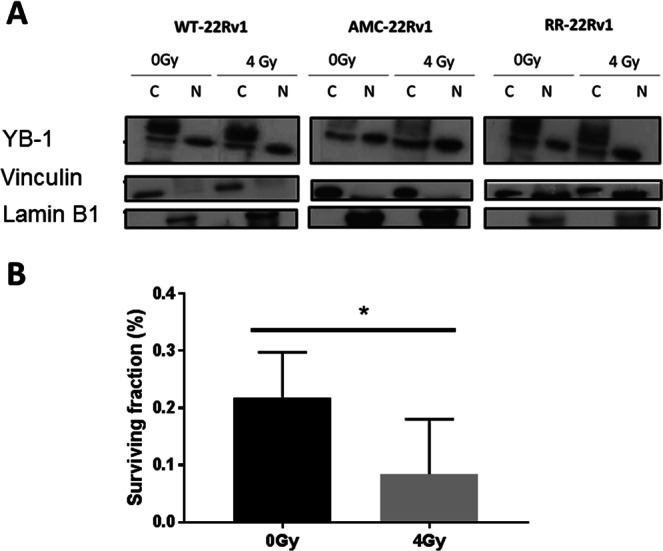
Evaluation of YB-1 as a marker of radioresistance. (**A**) Representative cropped immunoblots of YB-1 in the cytoplasmic (C) and nuclear (N) protein fractions of wild type (WT), age-matched controls (AMC) and radioresistant (RR) 22Rv1 prostate cancer cells which received a single dose of 4 Gy radiation or were left untreated. Vinculin and Lamin B1 were used as cytoplasmic and nuclear loading controls respectively. The full length blots are presented in Supplementary Fig. 6A–C. (**B**) Clonogenic survival of unirradiated and 4-Gy irradiated RR-22Rv1 cells treated with the YB-1 inhibitor Fisetin (60 μM, 24hrs). *p < 0.05.

**Figure 7:**
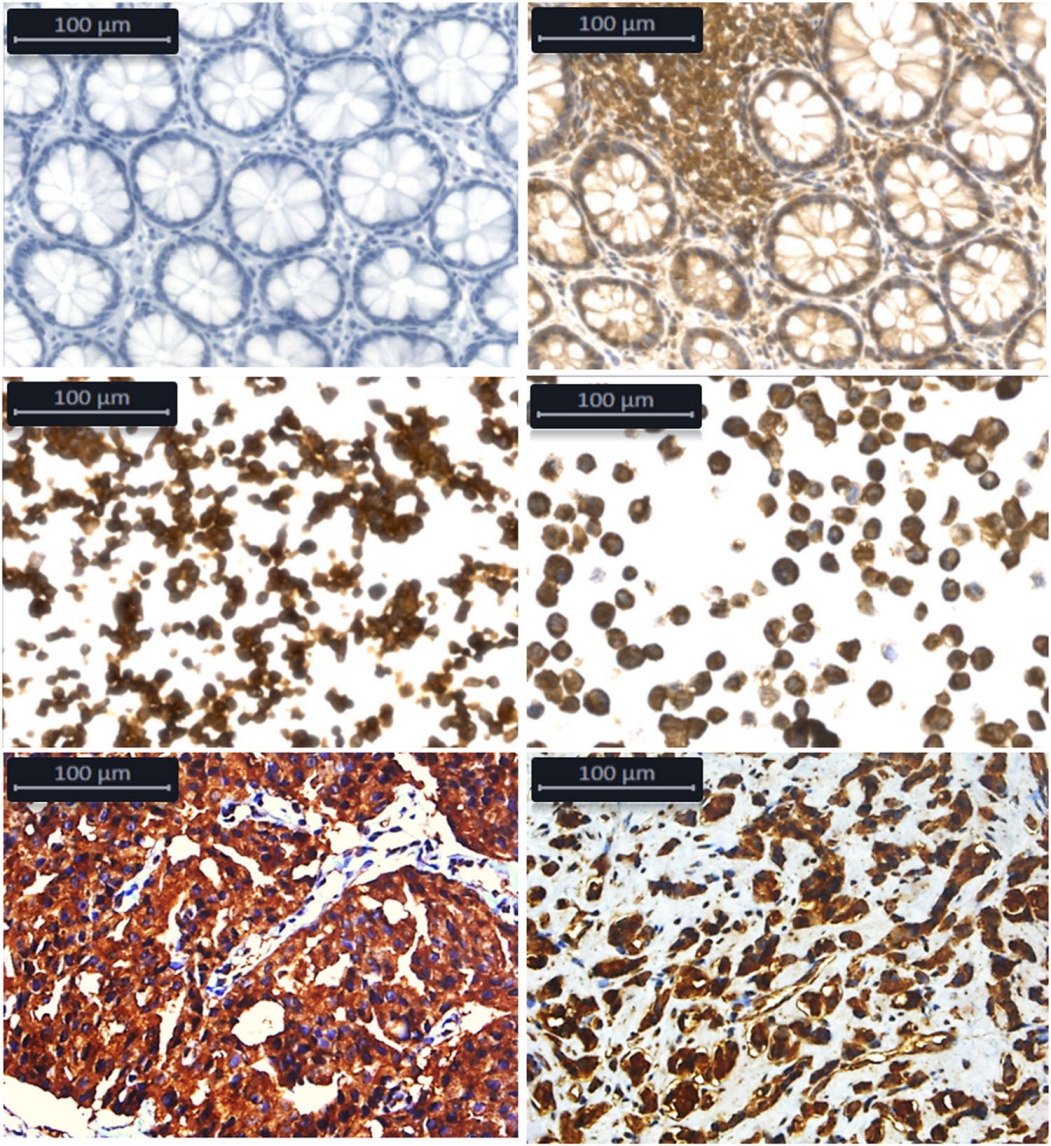
Representative images of positive and negative YB1 staining. Top left - Colon control tissue stained without primary antibody. Top Right - Colon control tissue displaying positive staining. Centre Left - WT-22RV1 cells exhibiting predominantly cytoplasmic staining for YB1. Centre right - RR-22RV1 cells which display cytoplasmic and enhanced nuclear YB1 staining. Bottom left - A pre-treatment prostate biopsy from a patient who subsequently underwent radiation therapy and exhibited a favourable response which display predominantly cytoplasmic YB1 staining. Bottom right - A biopsy from a non-responder/radioresistant patient displaying enhanced nuclear YB1 staining. Images were taken at 40X magnification using a Leica DM 3000 Microscope and Leica DFC 425 camera.

The independent validation of the expression of the YB-1 protein indicated strong cytoplasmic and nuclear expression in all three cell lines, with a slight increase in nuclear expression following 4Gy- irradiation (Fig. 6A). Immunostaining for YB-1 was next performed in a series of 22 formalin-fixed parafilm-embedded biopsy specimens from prostate cancer patients. Nuclear expression was detected in 3 out of 4 specimen obtained from patients diagnosed with biochemical failure following radiation therapy. Nuclear expression was not detected in any biopsy specimens from the patients in remission five years post treatment (n = 18) (Fig. 7). Treatment of RR-22Rv1 cells with the YB-1 inhibitor Fisetin (60 µM, 24 h) resulted in a significant reduction in surviving fraction in unirradiated and 4-Gy irradiated cells (Fig. 6D). The combination of Fisetin with radiation was associated with a reduction in clonogenic survival from 28.4% ± 4.4 (radiation alone) to 8.5% ± 5.1 (p < 0.05).

## Discussion

The effects of radiation exposure on cancer cells and subsequent regulation of cell fate has been linked to DNA damage induction^4,5^, all known cancer hallmarks^3^, and the tumour microenvironment^26^. This study proposed to compare the protein expression profiles for a selection of signaling proteins involved in these biological processes in a pre-existing isogenic model of 22Rv1 prostate cancer cells^23^. High content multiplex protein profiling identified twenty-three proteins within the selected panel whose expression was significantly modified in the radioresistant line, compared to the wild type cell line.

The DNA repair protein PARP*-*1 was independently identified as one of the protein whose expression was down-regulated in radioresistant cells, alongside the androgen receptor (AR). PARP-1 has been implicated in epithelial-mesenchymal-transition (EMT) during prostate tumorigenesis^27^, and its elevated expression was associated with poor prognosis following radiotherapy^28^. PARP-1 inhibition appears most effective in tumours with elevated PARP-1, but reduced AR and p53 expression levels^29,30^. Our data support these findings and suggest that tumour recurrence following exposure to fractionated radiation may result from the selection of cells with reduced PARP-1 and AR as well as elevated p53 expression. Further evaluation of this molecular combination in radiotherapy patients is warranted.

The expression of p53 was increased in RR-22Rv1 cells and correlated with the downregulation of miR-141. Reports of the upregulation of miR-141 in prostate cancer suggest its involvement in the metastatic process^31^, the regulation of AR activity^32^ and modifications in cell proliferation rates and apoptosis susceptibility^33^, but no previous reports have shown evidence of its involvement in the response to radiation therapy. Treatment of RR-22Rv1 cells with PFT-α, a known suppressor of p53 as well as glucocorticoid and heat shock receptor factor^34^ was associated with a very small but significant radiosensitisation effect. This is the first report of a radiosensitisation effect of this compound in prostate cancer and further evaluation of the magnitude of radiosensitisation that may be achieved is warranted.

Our data identifies the up regulation of the intracellular domain of the Notch-3 receptor in RR-22Rv1, which correlates with a mild radiosensitisation effect following exposure to the Notch inhibitor DAPT. Notch inhibitors are increasingly explored as novel anti-cancer strategies^35–37^ and their potential as radiosensitisers has been proposed^10^. We^38,39^, like others^40^, have previously reported the upregulation of the Notch-3 receptor in prostate cancer, and its role in hypoxic tumours^41^. Although Notch activity has been implicated in the radiation-induced stress response of prostate cancer cells^42^, no such strategy exists in prostate cancer. Exposure to a single 5Gy radiation dose led to undetectable expression of the intracellular domain of the Notch-3 receptor in wild type 22Rv1 cells. This loss did not correlate with a reduction in HES-1 expression, a known downstream target of the Notch pathway. This data indicates that the regulation of HES-1 is not dependent on Notch-3 activation. In prostate tumours, HES-1 elevated expression was also related to Notch-1 receptor activation^43^. In prostate cancer cells, while the selective genetic manipulation of members of the pathways (Notch-1^44^) impaired differentiation and tumour growth^45,46^, the pharmacological inhibition of the pathway with gamma-secretase inhibitors only caused mild modifications^47,48^. Examples of Notch targeted radiosensitising strategies are beginning to emerge in lung cancer^49^ and glioblastoma^42,50^.

Finally, the comparative analysis of these protein profiles identifies upregulation of the YB-1 protein. Nuclear localization of YB-1 has been proposed as an indicator of poor prognosis^51^, with high protein levels of YB-1 and MTA1 associated with a 3-fold increased risk for requiring future hormone therapy or radiation therapy in prostate cancer^21^. Our data confirmed detection of the protein in the cytoplasm and nucleus of all three cell lines, and a slight elevation in the RR-22Rv1 cells. Exposure of RR-22Rv1 cells to a 4 Gy single radiation dose resulted in a modest increase in nuclear YB-1 expression levels. Pilot analysis identified elevated nuclear expression in 3 out of 4 formalin-fixed-parafilm-embedded biopsy specimen from high risk radiotherapy prostate cancer patients whose tumours progressed post-treatment and none in those with no evidence of residual disease at five years. The inhibition of YB-1 by Fisetin was associated with microtubule stabilising properties, an ability to reverse mesenchymal features and inhibit cell proliferation, migration, and invasion^52^. Treatment of RR-22Rv1 with this dietary flanovol resulted in a significant reduction in the clonogenic survival of irradiated cells. This is the first report of the radiosensitisation potential of this YB-1 inhibitor.

In conclusion, the exposure of 22Rv1 prostate cancer cells to fractionated radiation and emergence of a radioresistant cell line is associated with the deregulation of signalling proteins associated with more aggressive disease and poor prognosis following radiotherapy. This work supports the clinical relevance of this model and identifies actionable markers of the radioresistant phenotype of prostate cancer.

## Materials and Methods

### Cell culture

Newly acquired, authenticated human 22Rv1 (WT-22Rv1) prostate cancer cells (American Type Culture Collection) were cultured at 37 °C in 95% humidified air containing 5% CO_2_ in RPMI cell culture medium containing l-glutamine (Lonza, Dublin, Ireland) with 10% foetal bovine serum (Gibco, Dublin, Ireland) and 1% pen/strep (Lonza). Fractionated 2 Gy X-rays doses (250 keV, 15 mA) using an RS225 cabinet (XStrahl, Surrey, UK) were delivered weekly to a cumulative dose of 60 Gy to generate the RR-22RV1 cell model, as described in^23^. Mock irradiated cells were cultured alongside to generate age-matched controls cells (AMC-22Rv1)^23^.

### Clonogenic assays

Cell survival was evaluated using a standard colony forming assay^53^. Cells were treated with Pifithrin-α (Sigma Aldrich, Dublin, Ireland) at concentrations of 20 µM and 40 µM for 2 hours; batimastat, DAPT or Fisetin (Sigma Aldrich) at a concentration of 60 µM for 24 hours. The cells were then irradiated at a single dose of 4 Gy and incubated for 7–14 days until formation of colonies. The colonies washed with PBS, were fixed and stained using a 0.05% Crystal Violet (Sigma Aldrich) in 70% Methanol solution. Individual colonies (defined as a cluster of greater than 50 cells) were counted using the ColCount software (Oxford Optronix Ltd, Abingdon,UK).

### DigiWest multiplex protein profiling

DigiWest analyses were performed as previously described^54^. Briefly, gel electrophoresis and Western blotting onto PVDF membranes was conducted using the NuPAGE system (Life Technologies, Carlsbad, USA). Blots were washed in PBST and proteins were biotinylated on the membrane using 50 µM NHS-PEG12-Biotin in PBST for 1 h, then washed in PBST and dried. Each sample lane was cut into 96 molecular weight fractions of 0.5 mm each and proteins were eluted in 96 well plates using per well 10 µl elution buffer (8 M urea, 1% Triton-X100 in 100 mM Tris-HCl pH 9.5). Eluted proteins from each molecular weight fraction were loaded onto one distinct color of neutravidin coated MagPlex beads (Luminex, Austin, TX, USA) and then pooled. A total of 10 µg of protein per cell sample was used for generation of 90 different antibody incubations.

Aliquots of the DigiWest bead mixes were added to 96 well plates containing 50 µl assay buffer (Blocking Reagent for ELISA (Roche, Rotkreuz, Switzerland) supplemented with 0.2% milk powder, 0.05% Tween-20 and 0.02% sodium azide). After discarding the assay buffer, 30 µl of diluted primary antibody was added per well. Following overnight incubation at 15 °C on a shaker, bead mixes were washed twice with PBST, and Phycoerythrin-labelled secondary antibodies (diluted in assay buffer) were added and incubated for 1 h at 23 °C. Beads were washed twice prior to readout on a Luminex FlexMAP 3D.

For quantification of antibody specific signals, the DigiWest analysis tool described in^54^ was used to identify peaks and to calculate peak areas. For comparative analyses, protein expression values were normalized to the total protein per lane.

### miRNA expression analysis

Total RNA was isolated as per the manufacturer’s instructions using the *mir*Vana™ miRNA Isolation Kit (Applied Biosystems, USA). RNA concentration was determined using the nanodrop 1000 spectrophotometer. Reverse transcription was carried out using the High Capacity cDNA Reverse Transcription Kit (Applied Biosystems) on the Gene Amp PCR System 9600 (Perkin Elmer). TaqMan RT-PCR was then performed in 384 well plates using the 7900HT Real-Time PCR System (Applied Biosystems) using primers and probes for miR-141, miR-1285 and miR-16 and TaqMan™ Universal PCR Master Mix, no AmpErase™ UNG (Thermo Fisher Scientific) mixed together with amplified cDNA samples as per the manufacturer’s protocols. MiRNA expression levels were calculated using the ΔΔCT method^55^ relative to the endogenous control, miR-16. A change in miRNA expression was considered significant if at least a 2-fold change (above 200% expression or below 50% expression) was observed, with a p value of ≤0.05 compared to WT-22Rv1 cells.

### Western blotting

Proteins were extracted from cells using either RIPA Lysis buffer (Santa Cruz, USA) or the NE-PER Nuclear and Cytoplasmic Extraction kit (Thermo Fisher Scientific, USA) modified with phenylmethanesulfonyl fluoride (PMSF) (200 mM), a protease inhibitor cocktail, and sodium orthovanadate (100 mM), according to the manufacturer’s instructions Protein concentration was determined using the Pierce™ BCA Protein Assay Kit ((Thermo Fisher Scientific). 20 µg of each protein sample was then resolved by SDS-PAGE on a 5% stacking gel and 10% resolving gel using a Mini-Protean Tetra Cell electrophoresis rig (BioRad, USA). Resolved proteins were then transferred to 0.2 µM Hybond PVDF membranes (Amersham, UK) using the XCell II™ Blot module (Invitrogen, Dublin, Ireland). Following transfer membranes were blocked using 5% w/v milk protein and probed using primary antibodies directed against PARP1 (#sc-8007; 1:100, Santa Cruz, UK^56^), AR (#sc-377546, 1:250, Santa Cruz), p53 (#sc-126, 1:500, Santa Cruz, UK^57^), Notch-3 (#ab23426, 1:250, Abcam, UK^58^), HES-1 (#ab71559, 1:1000, Abcam^59^) or YB-1 (#ab12148, 1:2000, Abcam^60^). After washing, the membrane was incubated with horseradish peroxidase conjugated goat secondary antibody (sc-2020, 1:1000, Santa Cruz^61^) or Anti-Rabbit IgG HRP Linked (#7074, 1:1000, Cell Signalling^62^) antibodies. Following incubation with the primary and secondary antibodies, a detection reagent luminol (SC-2048, Santa Cruz) was applied to blots and chemiluminescence images were then developed using a Fujifilm LAS-4000 luminescent image analyser. Molecular weight was confirmed using the MagicMark™ XP Western Protein Standard (LC5602, Thermo Fisher Scientific, Dublin, Ireland). Membranes were stripped prior to reprobing with either anti-actin (1:10000, Sigma-Aldrich^63^), anti-GAPDH (#ab9485, 1:10,000, Abcam^63^), Lamin (#GTX103292, 1:1,000, Genetex, UK^64^) or Vinculin (#GTX113294, 1:1,000, Genetex^64^).

### Patient specimens

Diagnostic biopsies (n = 22) were obtained retrospectively with informed consent from high-risk prostate cancer patients with a primary Gleason score of 8–10 who subsequently underwent androgen deprivation therapy and external beam radiation therapy. Ethical approval was obtained from St James’s/Tallaght Hospital and the St Luke’s Radiation Oncology Network, Dublin, Ireland. All research was performed in accordance with relevant guidelines/regulations. Four patients were diagnosed with biochemical failure and disease recurrence, according to the Phoenix criteria. The remaining patients (n = 18) were in remission at 5 years post- treatment.

### Immunohistochemistry

4 µM sections were cut from the 22 selected FFPE patient tissue blocks and were baked onto charged slides overnight in an oven at 65 °C prior to storage until needed. Slides were stained using the Rabbit specific HRP/DAB (ABC) Detection IHC Kit (Abcam, ab64261) and a rabbit polyclonal YB-1 antibody (Abcam, ab12148) diluted 1:100 in PBS. Immunostained sections were blindly examined by two trained histopathologists (SF & DC). Images were taken using a Nikon Eclipse E200 microscope and OC view 7 software.

### Statistical analysis

A Student’s t-test or one way ANOVA was used to compare means between treatment and control groups as appropriate. Statistical analysis was performed using Prism, Version 5.01 (GraphPad Software Inc. CA). A p-value of <0.05 was considered statistically significant.

### Supplementary information

Supplementary Figures

## Data Availability

The datasets generated during and/or analysed during the current study are available from the corresponding author on reasonable request.
