# Supplementary material for: Multiplex profiling identifies clinically relevant signalling proteins in an isogenic prostate cancer model of radioresistance
Source: Sci Rep. 2019 Nov 22;9:17325. doi: 10.1038/s41598-019-53799-7 (PMC6874565; doi:10.1038/s41598-019-53799-7)

Multiplex profiling identifies clinically relevant signalling proteins in an isogenic prostate cancer model of radioresistance

Inder S, [1,2] Bates M, [1] Ni Labhrai N, [1] McDermott N,[1] Schneider J, [3] Erdmann G,[3] Jamerson T,[4] Flores A,[4] Prina-Mello A, [5] Thirion P, [6] Manecksha P. R, [2,7] Cormican D, [8] Finn S,[8] Lynch T, [2] Marignol L[1*]

[1]Translational Radiobiology and Molecular oncology, Applied Radiation Therapy Trinity, Trinity Translational Medicine Institute (TTMI),Trinity College Dublin, Ireland

[2]Department of Urology, St James's Hospital, Dublin, Ireland

[3]NMI TT Pharmaservices, Berlin, Germany

[4]Department of International Health, Mount Sinai School of Medicine, New York, USA

[5]Laboratory for Biological Characterization of Advanced Materials (LBCAM), Trinity Translational Medicine Institute (TTMI), AMBER centre at CRANN Institute, Trinity College Dublin, Ireland; Department of Clinical Medicine, School of Medicine, Trinity College Dublin, Ireland.

[6] St Luke's Radiation Oncology Network, St James's Hospital, Dublin, Ireland

[7] Department of Surgery, Trinity College Dublin, Ireland

[8] Department of Histopathology, St James's Hospital, Dublin, Ireland

Supplementary Figure 1A: Full PARP-1 immunoblot of wild type (WT), age-matched controls (AMC) and radioresistant (RR) 22Rv1 prostate cancer. Three independent biolological replicates, noted as 1, 2, and 3 are presented. PARP-1 antibody, Santacruz, sc-8007, 1:100, 10s Exposure.

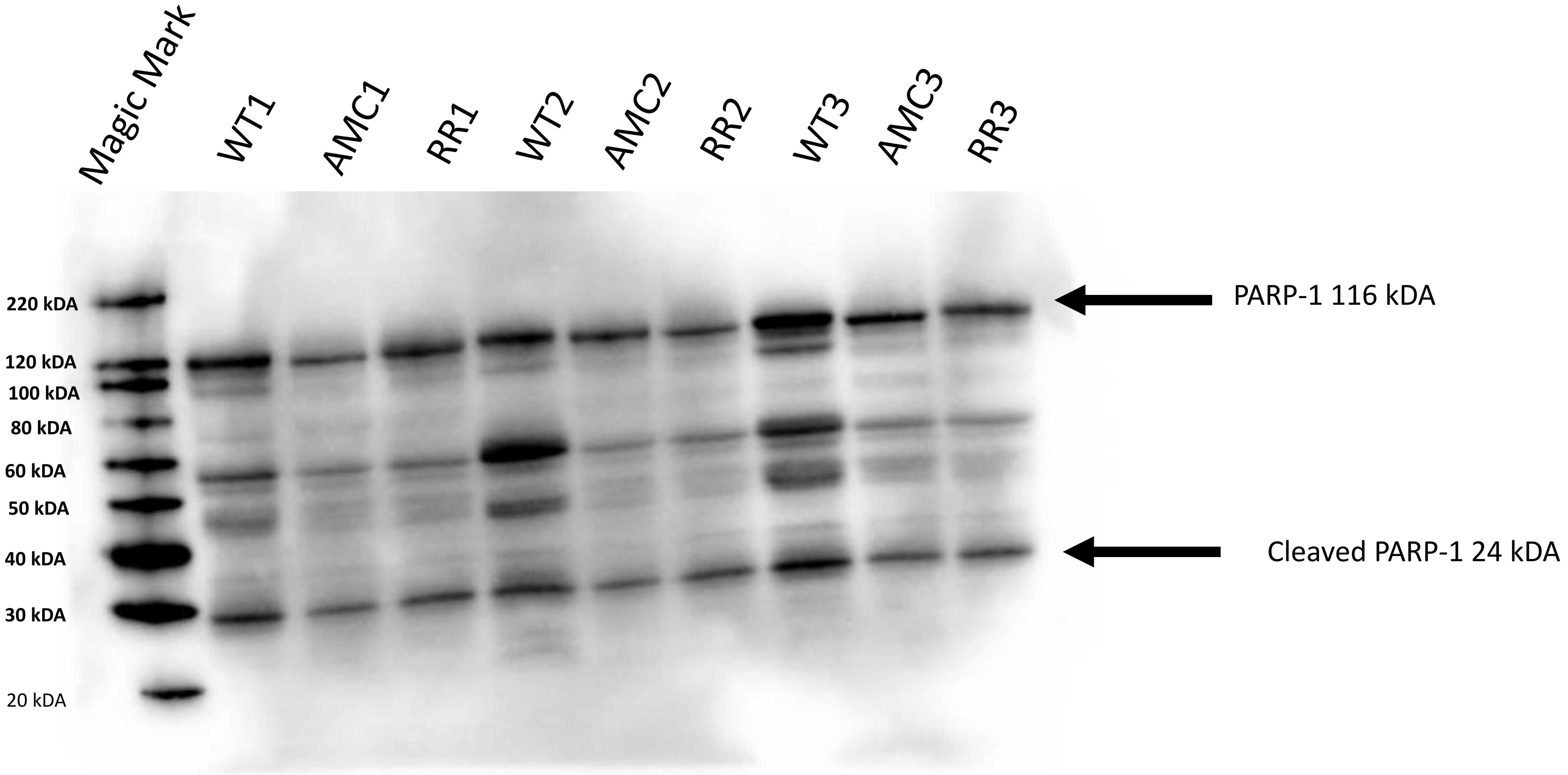

Supplementary Figure 1B: Full GAPDH immunoblot of wild type (WT), age-matched controls (AMC) and radioresistant (RR) 22Rv1 prostate cancer. Three independent biolological replicates, noted as 1, 2, and 3 are presented.

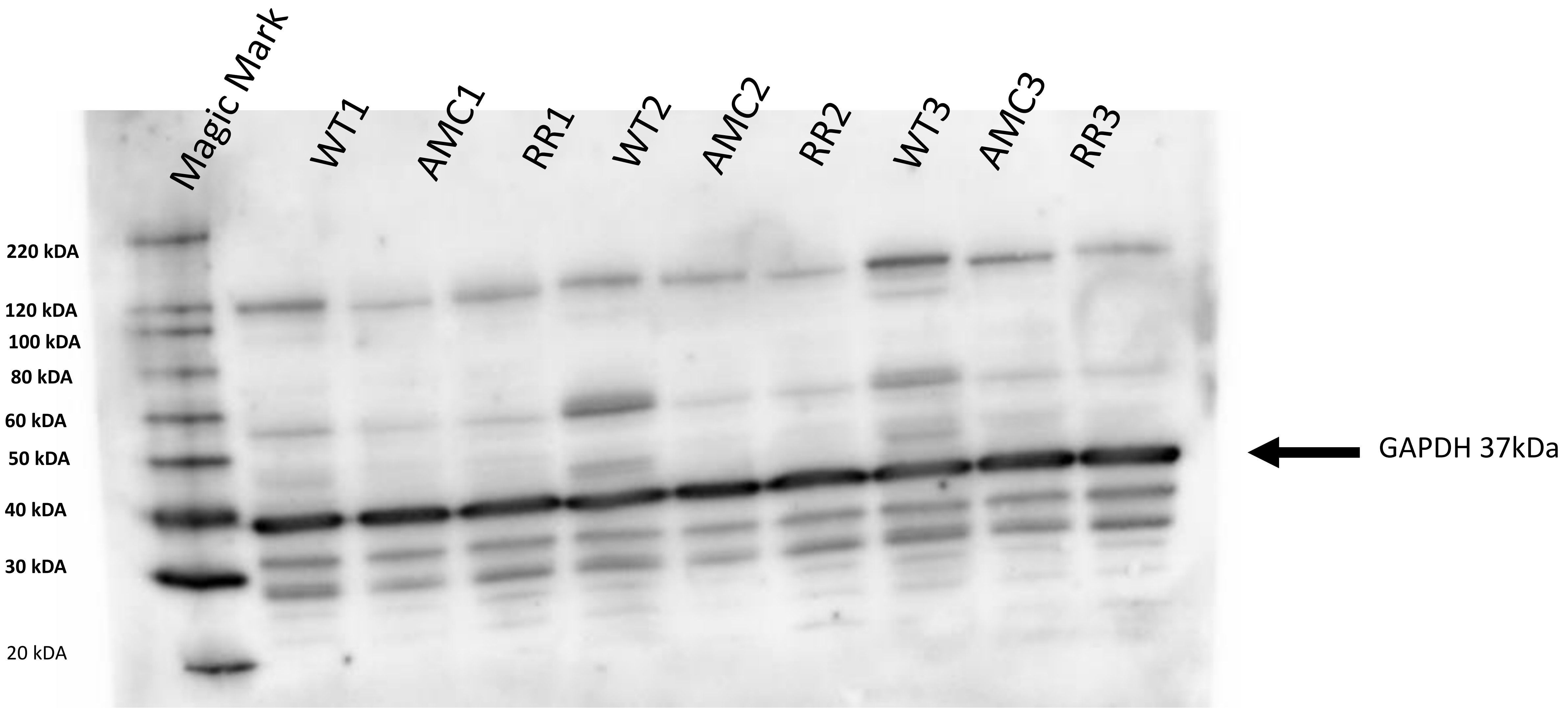

Supplementary Figure 2A Full Androgen Receptor (AR) immunoblot of the cytoplasmic (C) and nuclear (N) protein fraction of wild type (WT), age-matched controls (AMC) and radioresistant (RR) 22Rv1 prostate cancer.

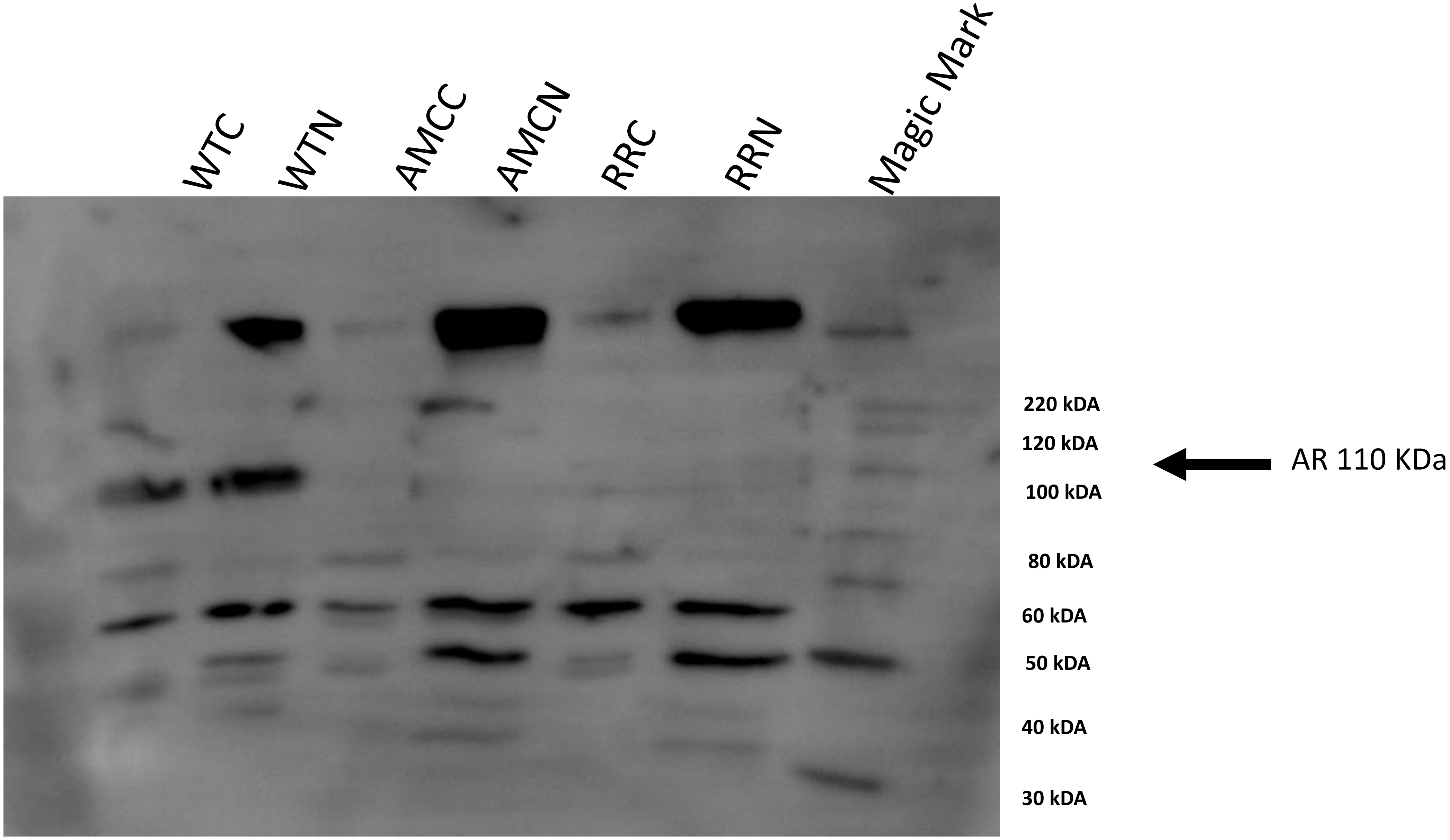

Supplementary Figure 2B Full Lamin immunoblot of the cytoplasmic (C) and nuclear (N) protein fraction of wild type (WT), age-matched controls (AMC) and radioresistant (RR) 22Rv1 prostate cancer.

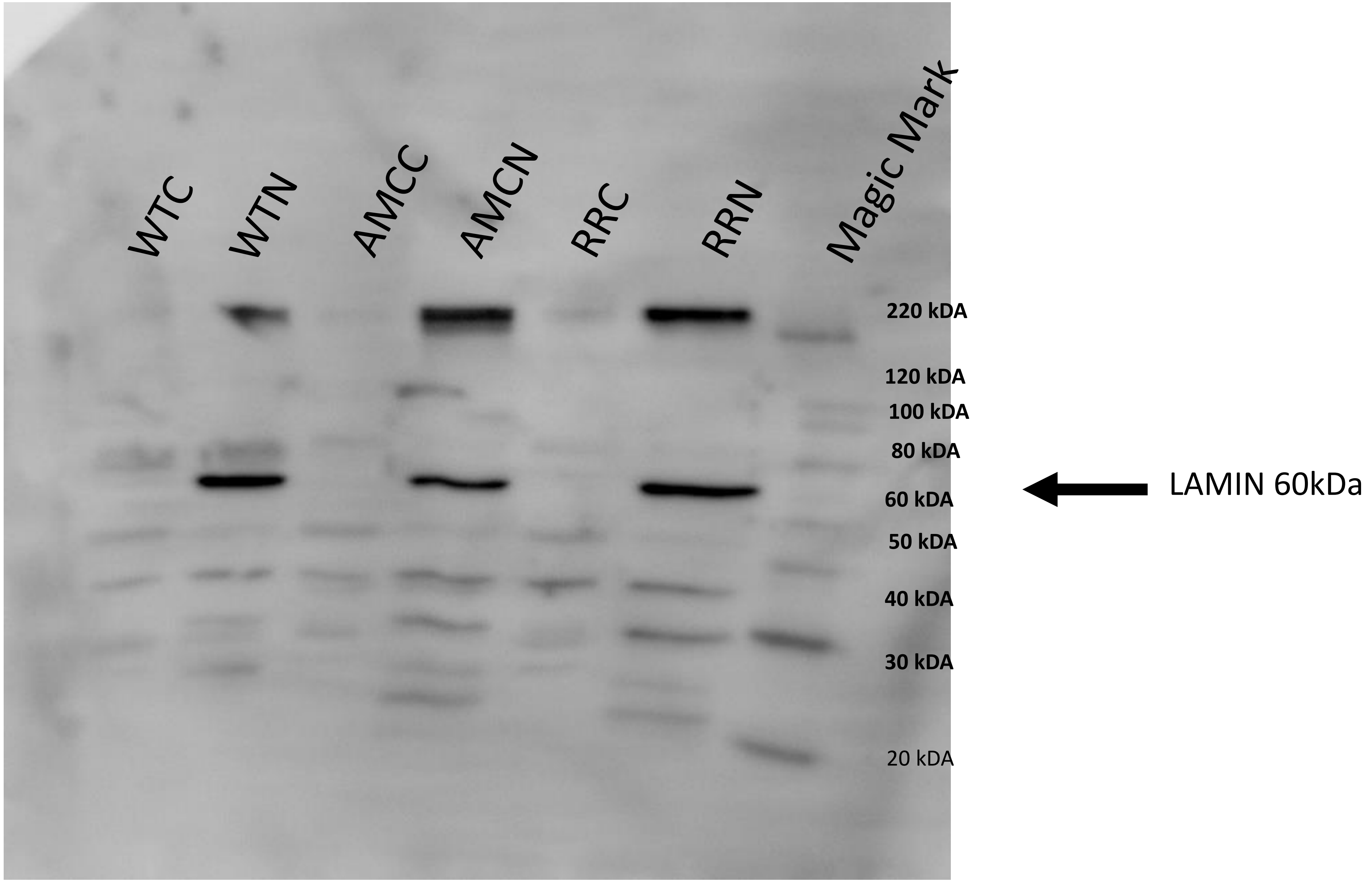

Supplementary Figure 2C Full GAPDH immunoblot of the cytoplasmic (C) and nuclear (N) protein fraction of wild type (WT), age-matched controls (AMC) and radioresistant (RR) 22Rv1 prostate cancer.

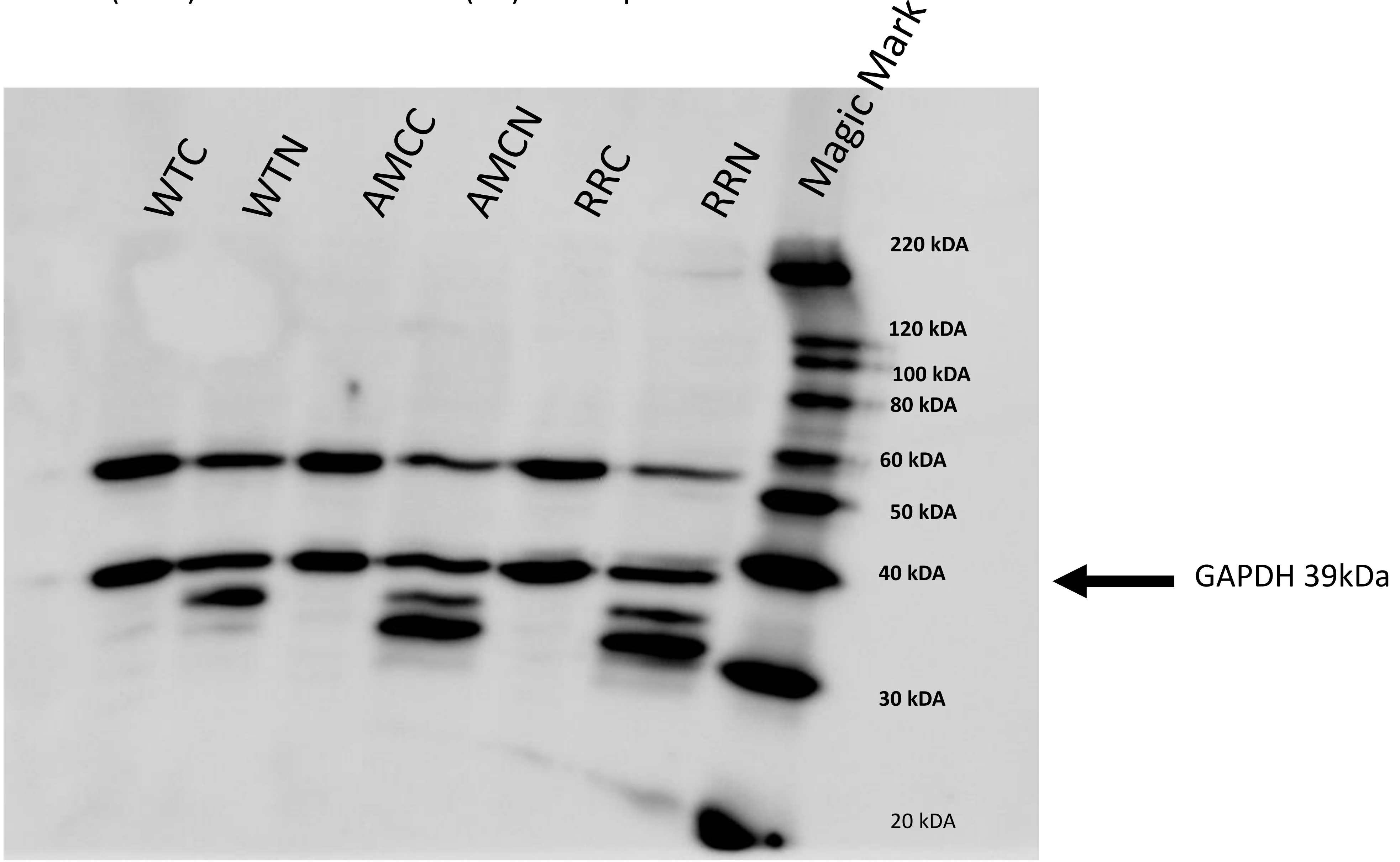

Supplementary Figure 3A: Full p53 immunoblot of wild type (WT), age-matched controls (AMC) and radioresistant (RR) 22Rv1 prostate cancer. Three independent biological replicates are presented.

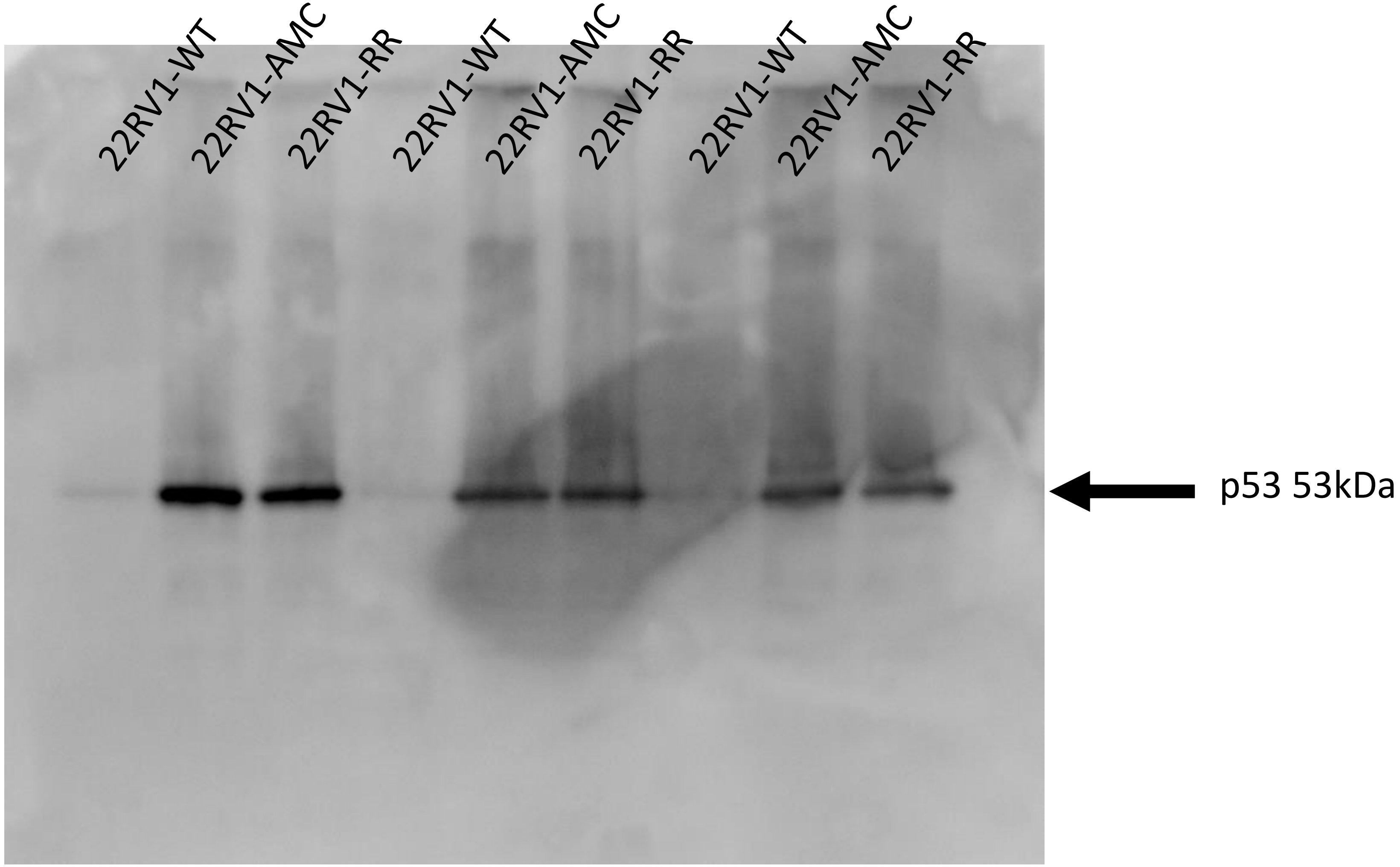

Supplementary Figure 3B: Full GAPDH immunoblot of wild type (WT), age-matched controls (AMC) and radioresistant (RR) 22Rv1 prostate cancer. Three independent biological replicates are presented.

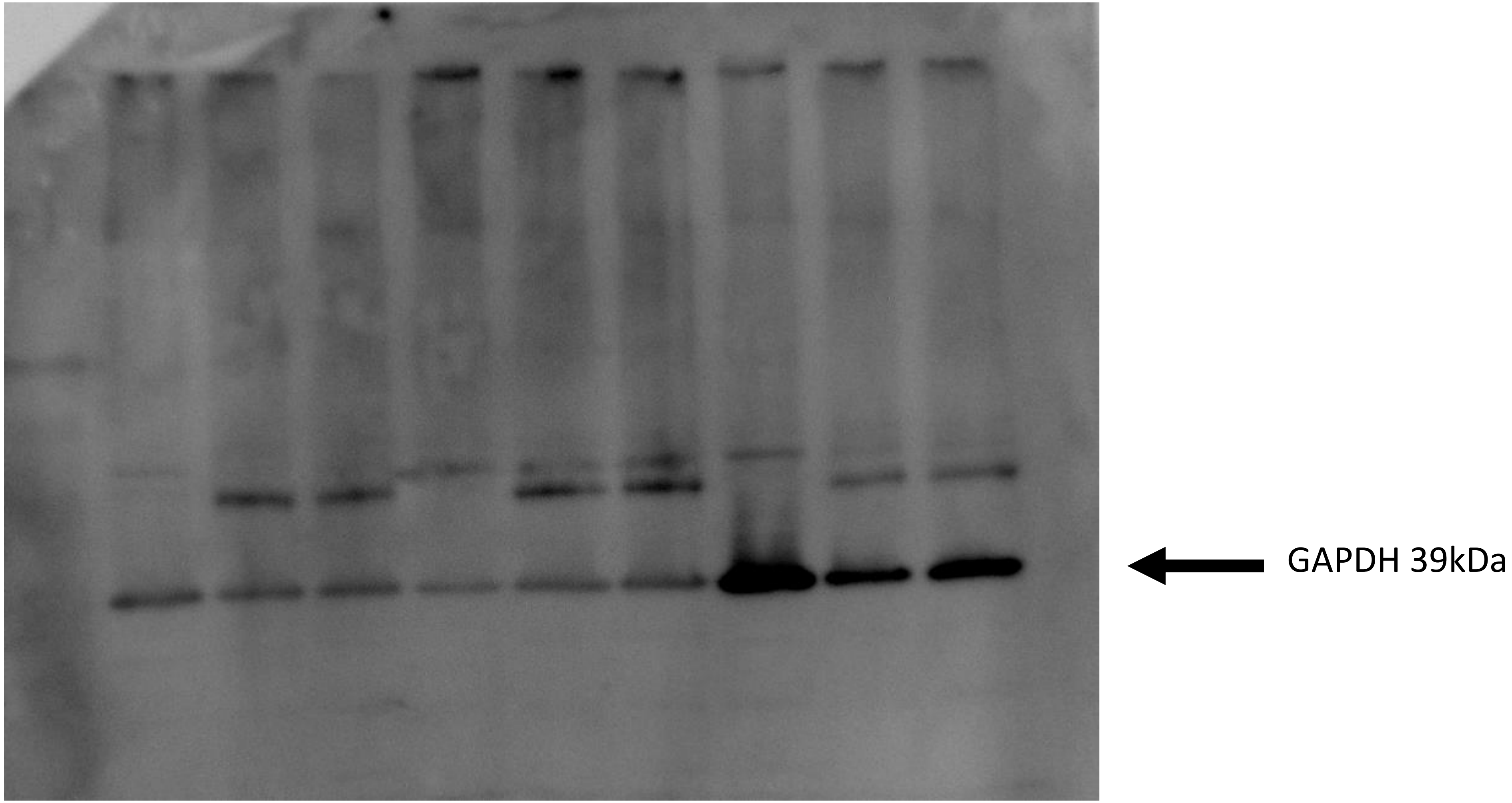

Supplementary Figure 4: Full Notch-3 and Actin immunoblot of wild type (WT), age-matched controls (AMC) and radioresistant (RR) 22Rv1 prostate cancer.

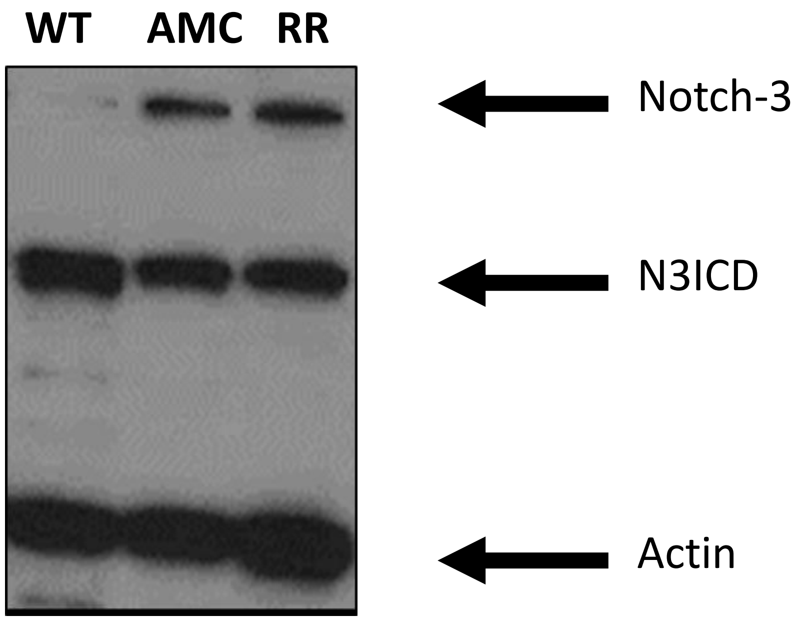

Figure 5A Full Notch-3 immunoblot of cell lysates of untreated (Controls) and 5Gy- irradiated 22Rv1, DU145 and PC3 prostate cancer cells.

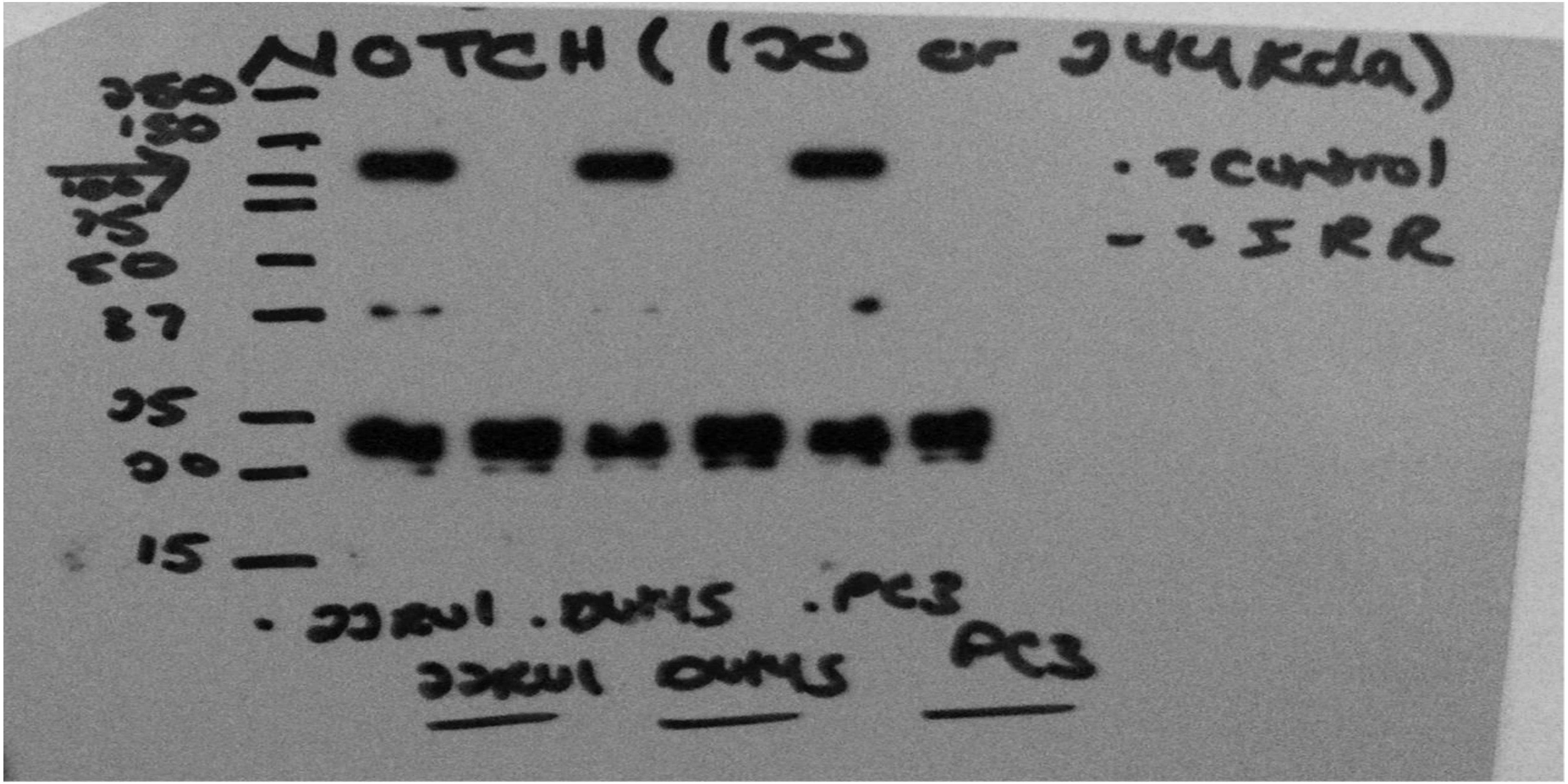

Figure 5B Full HES-1 immunoblot of cell lysates of untreated (Controls) and 5Gy- irradiated 22Rv1, DU145 and PC3 prostate cancer cells.

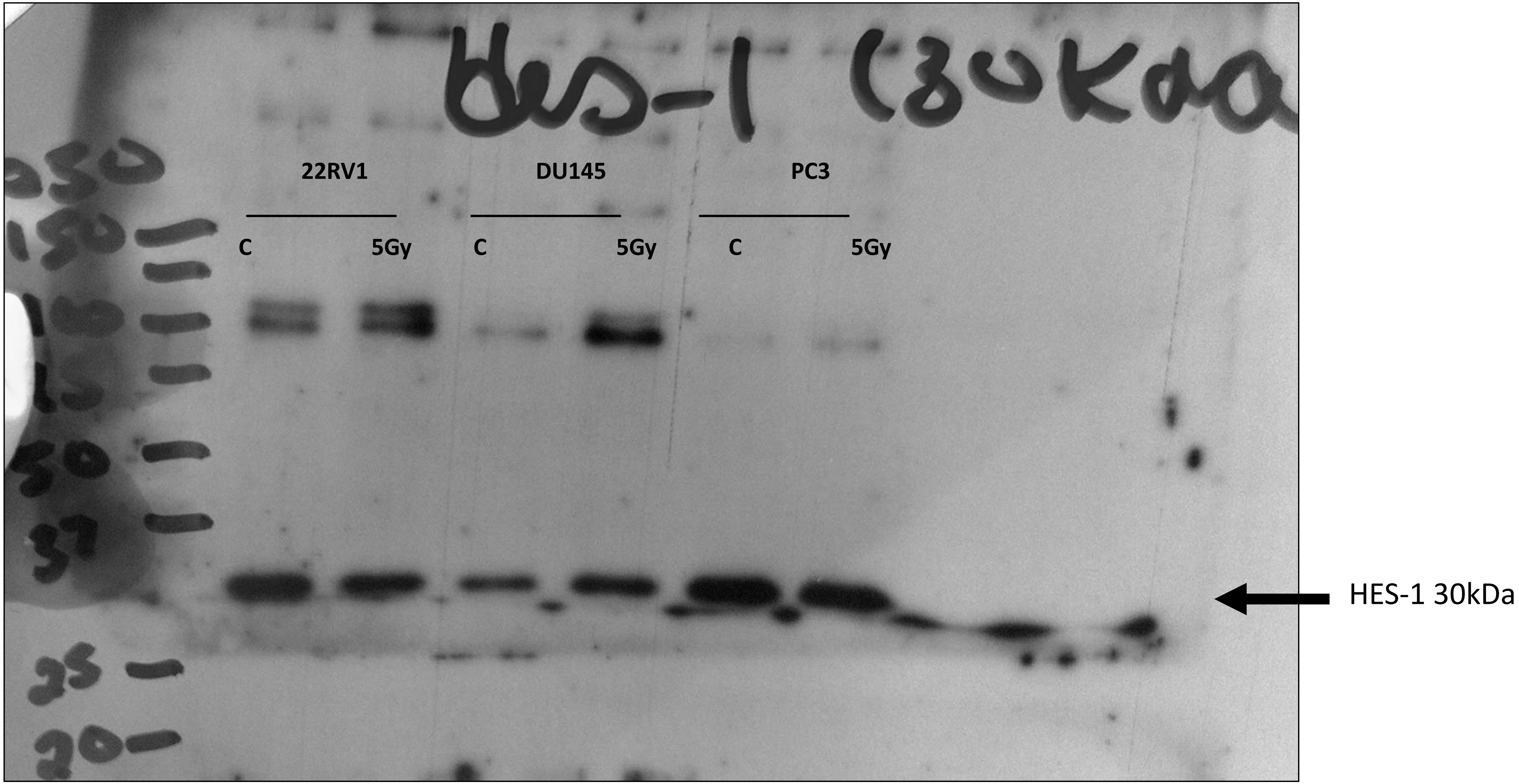

Figure 5C Full Tubulin immunoblot of cell lysates of untreated (Controls) and 5Gy- irradiated 22Rv1, DU145 and PC3 prostate cancer cells.

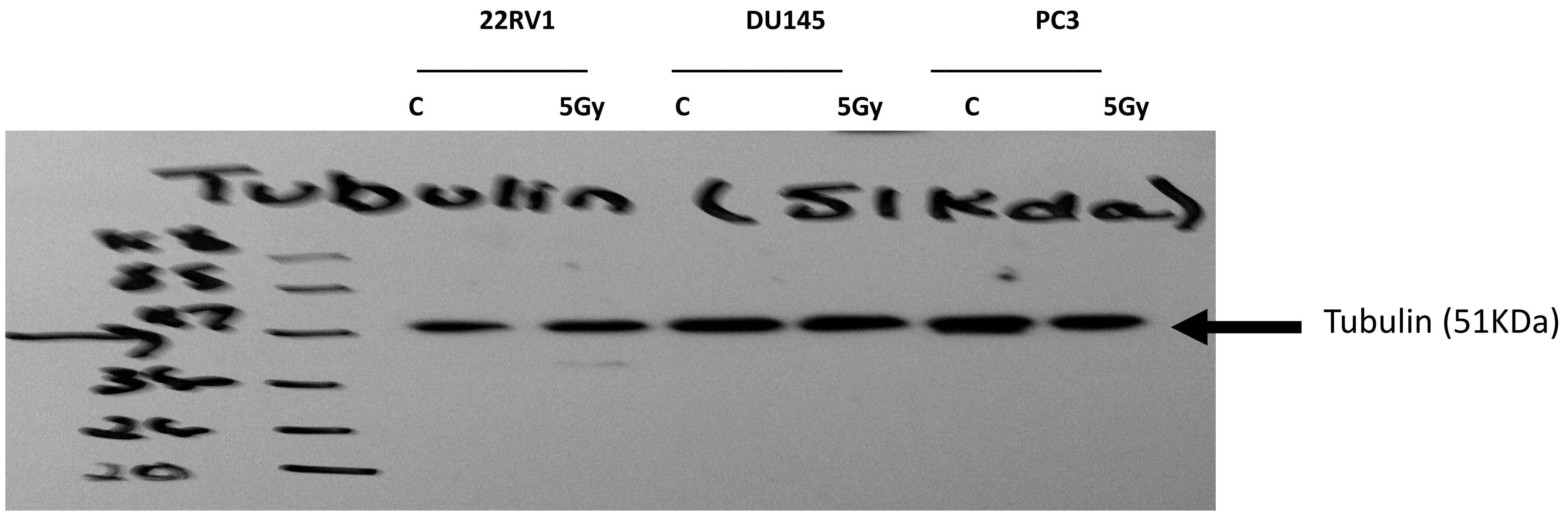

Supplementary Figure 6A: Full YB-1 immunoblot of wild type (WT), age-matched controls (AMC) and radioresistant (RR) 22Rv1 prostate cancer. Three independent biological replicates are presented. HeLa cell lysates were used as a control. MM, Molecular Marker.

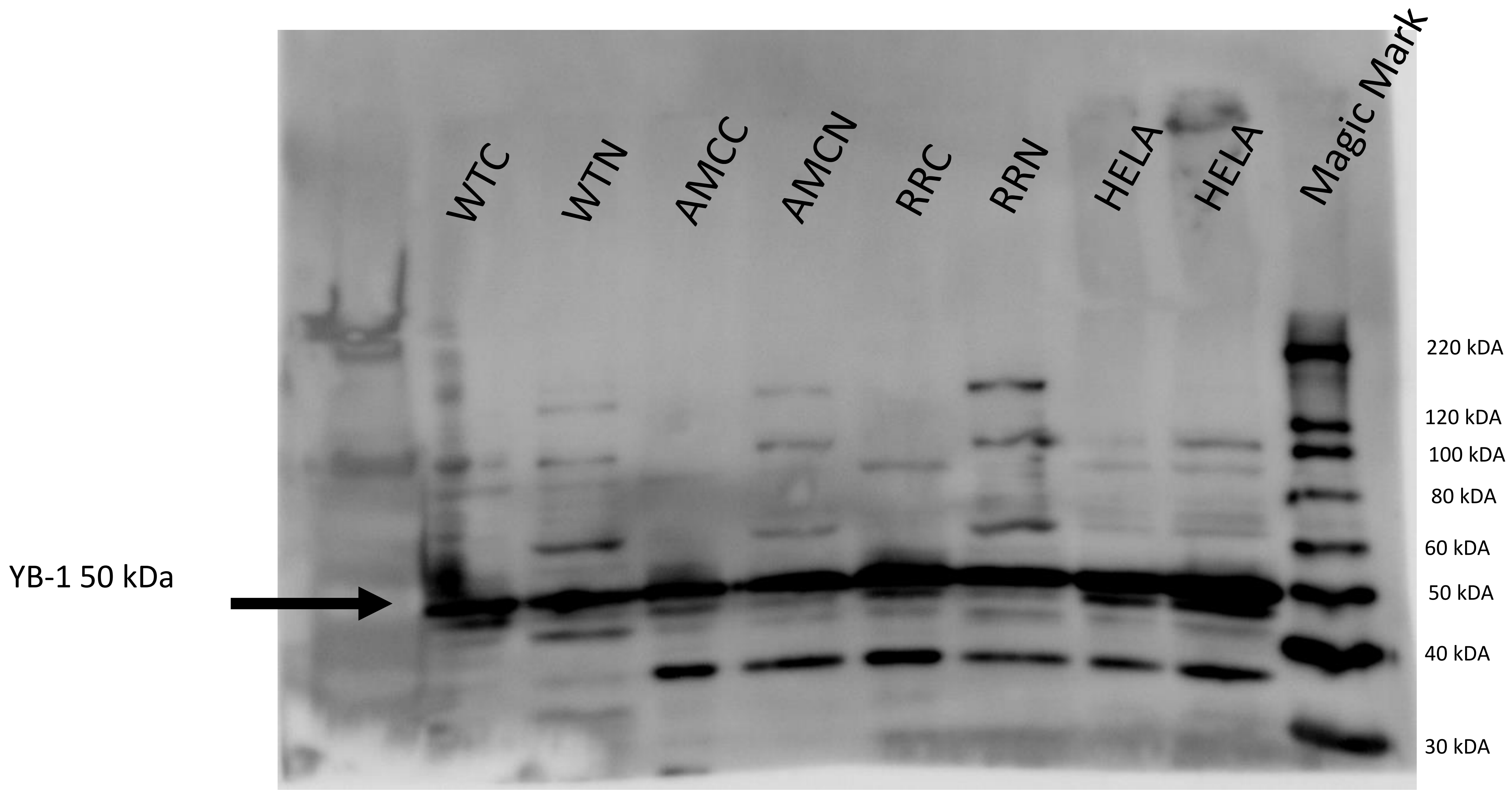

Supplementary Figure 6B: Full Vinculin immunoblot of wild type (WT), age-matched controls (AMC) and radioresistant (RR) 22Rv1 prostate cancer. Three independent biological replicates are presented. HeLa cell lysates were used as a control.

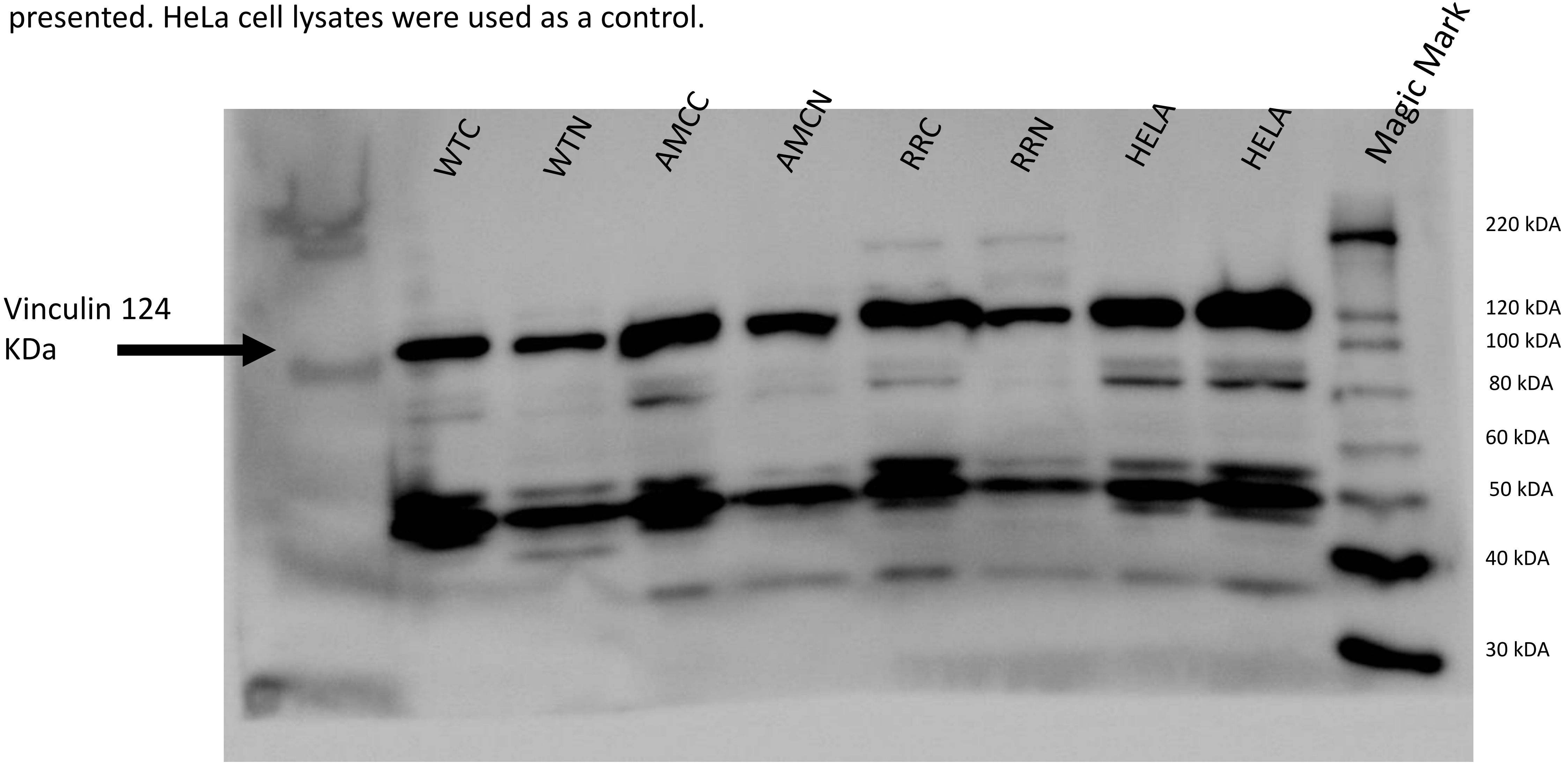

Supplementary Figure 6C: Full Lamin immunoblot of wild type (WT), age-matched controls (AMC) and radioresistant (RR) 22Rv1 prostate cancer. Three independent biological replicates are presented. HeLa cell lysates were used as a control..

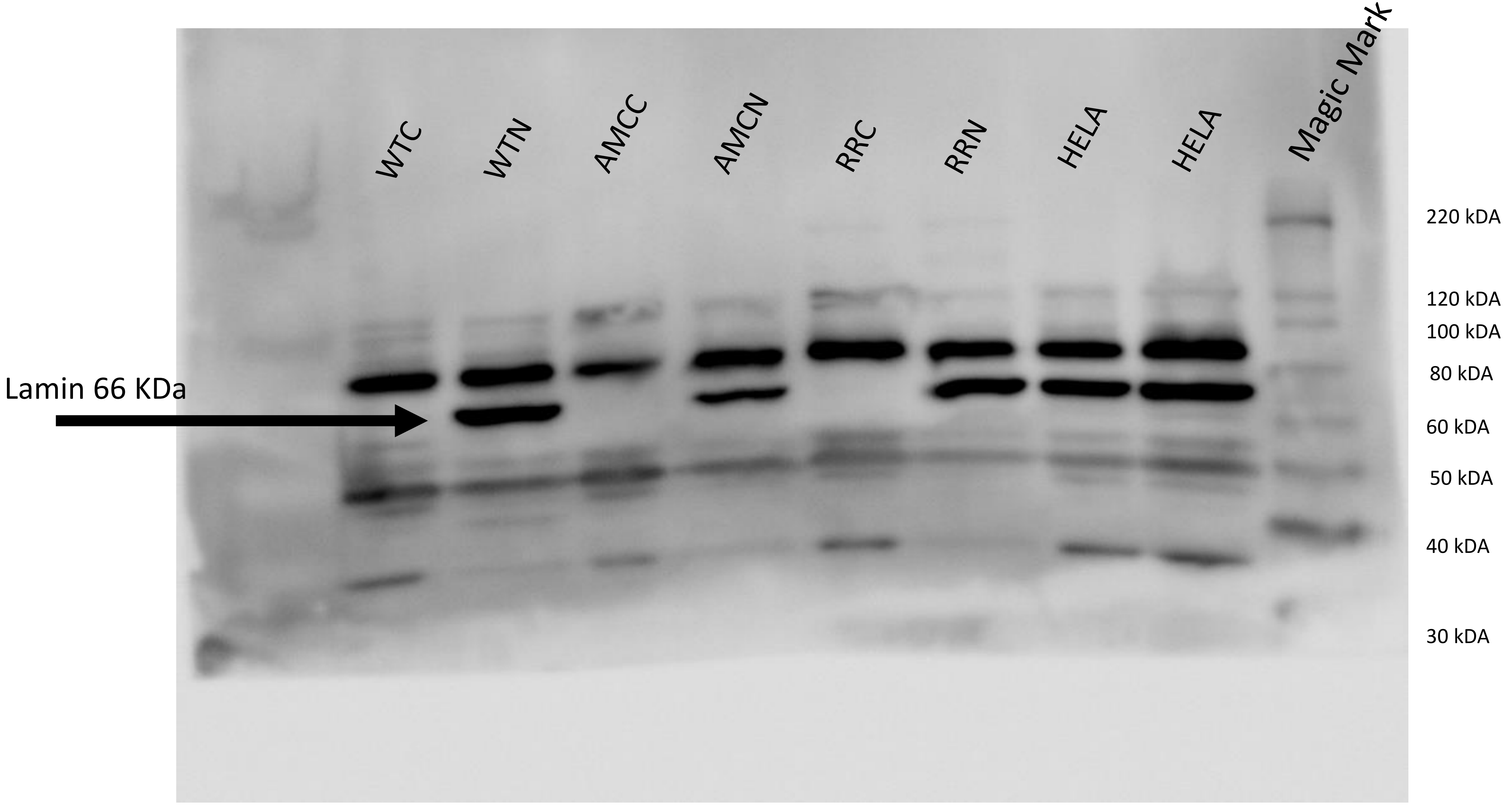

Supplement: Supplementary file 1 — Supplementary Figures [file 41598_2019_53799_MOESM1_ESM.pdf]
